# Molecular characterization of *Listeria monocytogenes* strains isolated from imported food in China from 14 countries/regions, 2003-2018

**DOI:** 10.3389/fcimb.2023.1287564

**Published:** 2023-12-20

**Authors:** Liying Zhu, Xuejiao Ji, Yuan Wu, Wei Xu, Feifei Wang, Xinxin Huang

**Affiliations:** ^1^Technical Center for Animal, Plant and Food Inspection and Quarantine of Shanghai Customs, Shanghai, China; ^2^Key Laboratory of Medical Molecular Virology (MOE/NHC/CAMS) and Shanghai Institute of Infectious Disease and Biosecurity, Shanghai Frontiers Science Center of Pathogenic Microorganisms and Infection, School of Basic Medical Sciences, Fudan University, Shanghai, China; ^3^Shanghai Clinical Research Center for Infectious Disease (tuberculosis), Shanghai Pulmonary Hospital, Tongji University School of Medicine, Shanghai, China

**Keywords:** *Listeria monocytogenes*, foodborne pathogen, cgMLST, WGS, SNP, virulence genes

## Abstract

*Listeria monocytogenes* (*Lm*) is associated with severe foodborne infections and ubiquitous in the nature. Identification of characteristics of *Lm* transmission through trading of food products is essential for rapidly tracking *Lm* sources and controlling dissemination of listeriosis. In this study, a total of 44 *Lm* strains were isolated from food products originating from 14 countries/regions during 2003-2018 at the Shanghai port. The genomes of these Lm strains were sequenced by high-throughput sequencing. Multilocus sequence typing (MLST) analysis showed that 43 isolates were divided into 17 sequence types (STs). The distribution of STs was decentralized, with the dominant ST2 accounting for only 18.18% of the strains. The LM63 strain did not match with any of the existing STs. Core-genome MLST (cgMLST) analysis based on 1748 core genes categorized the 44 strains into 30 cgMLST types (CTs), with CT10153 and CT7892 as the most predominant CTs. Notably, LM63 and LM67 shared the same CT in the cgMLST analysis. The phylogenetic analysis based on single-copy homologous genes revealed that the 44 *Lm* strains were primarily classified into two lineages. The SNP analysis also indicated that these strains were roughly divided into two clades, with strains in the first clade mainly collected earlier than those in the second clade, which were predominantly collected from 2010 onwards. The analysis using the virulence factor database (VFDB) indicated that the virulence gene *inlJ* was the most prevalent among these 44 strains. Notably, *ddrA*, *msbA*, and *sugC* were enriched in this dataset, requiring further clarification of their roles in *Listeria* through future studies. These results might provide a clue for understanding of the global epidemiology and surveillance of *Lm* and present insights for implementing effective measures to reduce or prevent *Listeria* contamination outbreaks in imported food products.

## 1 Introduction

*Listeria monocytogenes* (*Lm*), a facultative anaerobic Gram-positive bacterium, is a common foodborne pathogen ubiquitous in the nature (Bolívar and Pérez-Rodríguez, 2023). Upon infection of a susceptible host, *Lm* can cause a severe systemic infection, namely listeriosis, which can manifest as meningitis, sepsis, and even lead to abortion in pregnant women. Listeriosis is especially harmful to the elderly, pregnant women, children, newborns, and immunocompromised individuals(Koopmans et al., 2023). It is essential to recognize listeriosis outbreaks promptly and trace them to their food sources to prevent further infections.

Outbreak of listeriosis has been frequently reported in numerous countries worldwide (Moura et al., 2016; Halbedel et al., 2018; Shi et al., 2021), including China. For instance, listeriosis surveillance data collected from 2013 to 2017 revealed that 211 listeriosis cases were diagnosed in 64 sentinel hospitals of China (Li et al., 2019). Another study indicated that 155 *Lm* strains were isolated from different food products, such as ready-to-eat food, raw meat, raw poultry, and raw seafood in Shanghai (China) from 2009 to 2019 (Zhang et al., 2020). Recently, *Lm* contamination was identified in the ready-to-eat meat processing plants of two factories in Shanghai during 2019-2020 (Zhang et al., 2021a). These findings suggest that it is necessary to keep continuous monitoring and stringent surveillance of *Lm* strains due to the potential risk of listeriosis outbreak in Shanghai, which is a prestigious international trade center with a huge amount of ready-to-eat food import every year. However, the relationship between *Lm* carried by these imported foods and the outbreak of listeriosis in Shanghai has not been reported.

The pathogenicity of foodborne bacteria is closely associated with their antibiotic resistance and virulence (Banerji et al., 2021; Dubey et al., 2023). Strains that exhibit a strong antibiotic resistance can survive in unfavorable growth environments (Dharmasena et al., 2021). Besides, highly virulent strains can lead to more severe disease (Dubey et al., 2023). Accordingly, the antibiotic resistance and virulence of bacteria are mainly determined by the antibiotic resistance or pathogenic genes.

Multilocus sequence typing (MLST) analysis, one type of nucleotide sequence-based analysis methods concentrating on the single-nucleotide polymorphisms (SNPs) of *Lm* housekeeping genes, is widely utilized in the classification of *Lm* strains into different sequence types (STs) despite the low resolution (Maiden et al., 1998; Revazishvili et al., 2004). The results of epidemiological studies indicated that *Lm* strains with ST87 and ST8 were the most dominant types isolated from Chinese patients (Li et al., 2019), while *Lm* stain with ST9 was the most common type isolated from food (Wang et al., 2012; Zhang et al., 2020). Therefore, the MLST analysis of *Lm* strains may promote the identification of the relationship between *Lm* isolated from different sources, as well as determining sources of contamination. With the widespread extension of whole-genome sequencing (WGS), WGS-based high-resolution molecular subtyping technology has provided a significantly improved discrimination capability to identify the molecular characteristics of *Lm*. The core-genome MLST (cgMLST) and SNPs methods have been extensively applied in the investigation of listeriosis outbreak (Chen et al., 2016; Jackson et al., 2016; Moura et al., 2016). Consequently, the molecular typing of *Lm* isolated from food and clinical patients may promote exploration of crucial correlations between listeriosis cases and contaminated food sources. This, in turn, aids in the effective tracking of the origins of food contamination (Chen et al., 2017).

In the present study, food samples were collected from 14 different countries or regions over a span of 15 years, followed by isolation, and sequencing of 44 *Lm* genomes. Subsequently, the SNP mutation profiles of these strains were analyzed, a phylogenetic tree was constructed, and the tree was examined in the context of time and geographical location. The results revealed similarities among *Lm* strains from different regions at various time periods, suggesting transmission of these strains. These findings contribute to the understanding of the global epidemiology of listeriosis and underline the importance of monitoring of imported food products to prevent the outbreak of this disease.

## 2 Materials and methods

### 2.1 Collection of samples and bacterial DNA extraction

Among 49 isolates from 49 food samples collected from 14 countries or regions in East Asia (including China and Vietnam), Europe, North America, and South America during the period from 2003 to 2018, a total of 44 *Lm* strains were successfully identified at the Shanghai port of China. The remaining 5 samples were identified as contaminated with non-*Listeria* strains and were not used for further analysis. The isolated strains were obtained from samples of meat and chilled products that were imported from Shanghai Port. The demographic characteristics of the isolated strains are listed in **Table 1**.

**Table 1:** The demographic characteristics of 44 *L. monocytogenes* isolated strains.

Isolates	Source	Countries/regions	Year	MLST/ST	*abcZ*	*bglA*	*cat*	*dapE*	*dat*	*ldh*	*lhkA*
LM68	Bovine Fore Tendon	Switzerland	2013	2	1	1	11	11	2	1	5
LM74	Pangasius Fillet	Vietnam	2014	2	1	1	11	11	2	1	5
LM76	Pangasius Fillet	Vietnam	2015	2	1	1	11	11	2	1	5
LM1801	Frozen Pangasius Fillet	Vietnam	2018	2	1	1	11	11	2	1	5
LM1802	Frozen Pangasius Fillet	Vietnam	2018	2	1	1	11	11	2	1	5
LM1803	Frozen Pangasius Fillet	Vietnam	2018	2	1	1	11	11	2	1	5
LM1804	Frozen Pangasius Fillet	Vietnam	2018	2	1	1	11	11	2	1	5
LM1807	Frozen Pangasius Fillet	Vietnam	2018	2	1	1	11	11	2	1	5
LM70	Chicken Wings	Brazil	2014	3	4	4	4	3	2	1	5
LM67-1	Pig Face	France	2013	3	4	4	4	3	2	1	5
LM66	Pig Face	France	2013	4	1	2	12	3	2	5	3
LM84	Airlines Food (Sandwich)	Italy	2017	5	2	1	11	3	3	1	7
LM7	Frozen Chicken Wings	USA	2005	5	2	1	11	3	3	1	7
LM75	Frozen Chicken Drumsticks	USA	2015	5	2	1	11	3	3	1	7
LM65	Chicken Wings	Brazil	2013	9	6	5	6	4	1	4	1
LM103	Thigh Strips	China	2010	9	6	5	6	4	1	4	1
A198	Raw delicious bacon slices	Shanghai,China	2014	9	6	5	6	4	1	4	1
A195	Raw Unsalted Butter	Shanghai,China	2014	9	6	5	6	4	1	4	1
LM69	Pig Ribs	UK	2014	9	6	5	6	4	1	4	1
LM83	Atlantic Salmon	Chile	2016	31	7	14	10	19	9	8	1
LM80	Salmon	Chile	2016	31	7	14	10	19	9	8	1
LM64	Pig Ear	Germany	2013	37	5	7	3	5	1	8	6
LM60	Smoked Salmon	China	2012	87	12	1	4	14	3	39	4
LM72	Chilled Atlantic Salmon	New Zealand	2014	87	12	1	4	14	3	39	4
LM61	Frozen Salmon	Norway	2012	87	12	1	4	14	3	39	4
LM62	Frozen Salmon	Norway	2012	87	12	1	4	14	3	39	4
A248	Semi-finished egg fillings	Shanghai,China	2014	87	12	1	4	14	3	39	4
A189	Tuna Grain Sandwich	Shanghai,China	2014	87	12	1	4	14	3	39	4
A224	The ground below the link production line 1	Shanghai,China	2014	87	12	1	4	14	3	39	4
LM77	Salmon	Canada	2015	91	7	6	15	6	5	2	1
A213	Mango Chicken Roll	Shanghai,China	2014	101	7	15	15	8	6	14	9
A216	Bacon Egg Sandwich	Shanghai,China	2014	101	7	15	15	8	6	14	9
A277	Trash bin at the end of the assembly line belt	Shanghai,China	2014	101	7	15	15	8	6	14	9
LM1805	Frozen Chicken Wings	Brazil	2018	121	7	6	8	8	6	37	1
LM1808	Atlantic Salmon	Canada	2018	121	7	6	8	8	6	37	1
LM102	Salmon	Norway	2010	121	7	6	8	8	6	37	1
A257	Door handle inside the entrance of the workshop	Shanghai,China	2014	155	7	10	16	7	5	2	1
LM2	Frozen Chicken Three- section Wings	USA	2003	155	7	10	16	7	5	2	1
LM82	Atlantic Salmon	Canada	2016	296	12	12	12	61	3	1	4
LM5	Frozen Chicken Feet	Brazil	2005	297	5	42	5	7	6	2	1
LM71	Pig Head	Denmark	2014	403	7	7	10	4	5	24	1
LM78	Salmon	Chile	2015	426	11	1	48	3	2	1	31
LM3	Frozen Fries	USA	2003	648	5	29	102	62	6	7	34
LM63	Smoked Salmon	Norway	2012	NEW	4	4	4	3	NEW	1	5

Their sequence types were detected by multi-site sequence typing (MLST) based on housekeeping genes of *abcZ*, *bglA*, *cat*, *dapE*, *dat*, *ldh* and *lhkA*.

Before collecting samples, the surface of food bags was cleaned according to the aseptic operation protocols. About 25 g of each sample was collected and put in a homogenizing cup, containing 225 mL of LB1 enrichment solution under aseptic operation, and samples were homogenized at 8000~10000 r/min for 1~2 min, followed by incubation at 30°C for 24 h. Next, 0.1 mL suspension was transferred to 10 mL of LB2 enrichment solution, and incubated at 30°C for 24 h. Next, LB2 solution was seeded onto the *Listeria* chromogenic agar plate (Cat No: NCM1004; Neogen, New York, USA), and incubated at 36°C for 24~48 h. Subsequently, 3-5 colonies were collected from each plate and identified using Vitek Compact 2 kit (Meriere Biological Co., Ltd., Paris, France), which is an automatic biochemical analyzer. All isolated strains were stored at -80°C. DNA from each strain was extracted via a bacterial genome extraction kit according to the manufacturer’s instructions (Qiagen, Hilden, Germany), followed by storing at -20°C for further sequencing.

### 2.2 WGS, assembly and evaluation

To perform WGS, DNA quality and concentration were initially evaluated using the Qubit dsDNA HS Assay kit (Thermo Fisher Scientific, Waltham, MA, USA). Subsequently, the paired-end genomic libraries of each isolate were constructed, and sequencing was carried out on an Illumina instrument in PE250 mode. The raw reads were trimmed, draft genomes were then assembled using SOAPdenovo (Ver. 2.04) system (Van Der Zwan et al., 2018), and the gap-filling and base correction of the assembly genomes were carried out using GapCloser (ver. 1.12) system (Xu et al., 2020). The assembly results were comprehensively evaluated according to the overall length of the scaffolds (ranging from 2884308 to 3119043 bp), the number of scaffolds (ranging from 9 to 38), and the scaffold N50 (ranging from 215544 to 1551995 bp). Finally, the optimal genome assembly results were selected according to the K-mer values.

### 2.3 MLST and cgMLST analysis

The housekeeping genes for *Lm* (*abcZ*, *bglA*, *cat*, *dapE*, *dat*, *ldh* and *lhkA*) were utilized for MLST profiles of all isolated strains (Ragon et al., 2008; Carroll et al., 2017). The assembled genomes were uploaded to the MLST database of *Lm* (https://pubmlst.org/multilocus-sequence-typing) to determine MLST profiles (Maiden et al., 1998; Jolley et al., 2018). The alleles of the 7 loci of these strains were obtained, and the ST genotypes of these strains were identified according to the number of nucleotide differences between alleles.

In cgMLST analysis, *Lm* GCA_9001872251.1 (GenBank Accession No. LT906436.1) was utilized as a reference gene, and the best cgMLST values of each sample were matched according to the cgMLST1748 database (https://bigsdb.pasteur.fr/listeria/listeria.html) (Moura et al., 2016, Zhang et al., 2021b). Samples with a locus detection rate greater than 97% were included in the subsequent cgMLST typing analysis. Finally, these 44 *Lm* strains were divided into 30 CTs, including CT6150, CT3102, CT9387, CT5746, CT4677, CT7735, CT12277, CT10, CT3014, CT9470, CT7892, CT10153, CT6244, CT14053, CT2053, CT13475, CT96, CT11266, etc. A minimum spanning tree was generated using PHYLOViZ based on the results of cgMLST (Francisco et al., 2012).

### 2.4 Phylogenetic analysis

For the SNP-based phylogenetic tree construction, using GCA_9001872251.1 as the reference sequence, the trimmed reads were aligned by the Nucmer (Ver. 4.0.0) (Leekitcharoenphon et al., 2012). The SNPs, including DNA sequence polymorphisms caused by single-based conversion, transversion, and insertion/deletion, of each isolated strain were called with VarScan (Ver. 2.3) (Koboldt et al., 2009). Besides, SNPs with low sequencing depth and alignment quality values were filtered out. Subsequently, the matrix of 34,128 SNPs from all strains with reference alleles was generated, and the phylogenetic tree was constructed using the FastTree (Ver. 2.0.0) (Price et al., 2009).

For single-copy gene-based phylogenetic tree construction, single-copy homologous genes from homologous gene analysis were selected for multiple sequence alignment using MAFFT (http://mafft.cbrc.jp/alignment/software/) (Ver. 7.0) (Katoh and Standley, 2013), and alignment quality control with Gblocks (http://molevol.cmima.csic.es/castresana/Gblocks.html) (Ver. 0.91b) (Talavera and Castresana, 2007). Finally, the single-copy gene phylogenetic tree was inferred using RAxML (https://github.com/stamatak/standard-RAxML) (Ver. 8.0) (Stamatakis, 2014).

### 2.5 Analysis of mobile genetic elements

To identify MGEs in *Listeria*, it was attempted to analyze the number of prophages, clustered regularly interspaced short palindromic repeats (CRISPRs), and insertion sequences (ISs) in genome DNA sequence of isolated strains. Prophages were predicted in the genomes of 44 strains using the PHASTER database (Arndt et al., 2016). According to the predictive values, the prophages were classified into three categories: predictive value < 70, incomplete prophage; 70 ≤ predictive value < 90, questionable prophage; 90 ≤ predictive value, intact prophage. The numbers of all prophages and intact prophages are displayed in **Appendix Table 1**. CRISPRs were predicted using Minced (Ver. 0.2.0; https://sourceforge.net/projects/minced/) in isolated samples (Moller and Liang, 2017). The relevant statistics are presented in **Appendix Table 2**. ISs were predicted through Basic Local Alignment Search Tool (BLAST) (evaluate = 1-5) and ISfinder databases (Siguier et al., 2006). Furthermore, ISs were predicted in all 44 samples, while only 18 samples had complete ISs. The number of ISs is listed in **Appendix Table 3**.

### 2.6 Analysis of virulence genes

The virulence factor genes of 44 *Lm* strains were identified using the Virulence factors database (VFDB) (Chen et al., 2005), and confirmed by BLAST to determine the virulence of each strain. Heatmap and hierarchical clustering graph of gene distribution were drawn according to the abundance of virulence factor genes in each isolated strain, and samples and virulence factors were clustered using the pheatmap R package (Ver. 1.0.12). Samples illustrating similar distribution patterns of different virulence factors were grouped close to each other on the clustering tree. Moreover, the origin region of sample, isolation time, and cgMLST classification were marked on the map.

## 3 Results

### 3.1 Characteristics of bacterial strains

To analyze the genotype characteristics and virulence factors of bacteria isolated from 49 food samples collected from 2003 to 2018, the genomic DNA was sequenced using high-throughput sequencing. Among them, 44 isolates were identified as *Lm*, 2 isolates were identified as *Enterococcus faecalis*, and the other three were mixtures of various bacteria. Isolates identified as *Enterococcus faecalis* and those that were mixtures of various bacteria were excluded from subsequent analyses.

The demographic characteristics of 44 *Lm* strains are summarized in **Table 1**. Briefly, 11 strains were isolated from food products and food-processing environments in China, 7 strains were isolated from frozen food products imported from Vietnam, 4 strains from Brazil, the United States, and Norway, 3 strains from Canada and Chile, 2 strains from France, and the remaining 6 strains from Denmark, Germany, Switzerland, New Zealand, Italy, and the United Kingdom, respectively (**Table 1**).

To identify the genetic and evolutionary relationships between these *Lm* strains, MLST genotyping analysis was performed on the 44 isolated strains. The results showed that the 43 isolates were classified into 17 STs. The distribution of ST types was relatively dispersed. Specifically, ST2 was the most prevalent type, representing only 18.18% (8/44) of the strains. This was followed by ST87 (7/44, 15.91%), ST9 (5/44, 11.36%), with three occurrences each of ST5, ST101, and ST121, and two instances each of ST3, ST31, and ST155. The remaining ST types, including ST4, ST37, ST91, ST296, ST297, ST403, ST426, and ST648, each appeared only once (**Table 1**). Furthermore, the last strain, LM63 achieved from Norway in 2012, could not be classified as any of the existing STs because of an unknown genotype in the *dat* gene containing a new SNP, demonstrating that the mentioned strain might have a temporarily unknown activity or function.

### 3.2 cgMLST analysis of *L. monocytogenes* isolates

**Figure 1:**
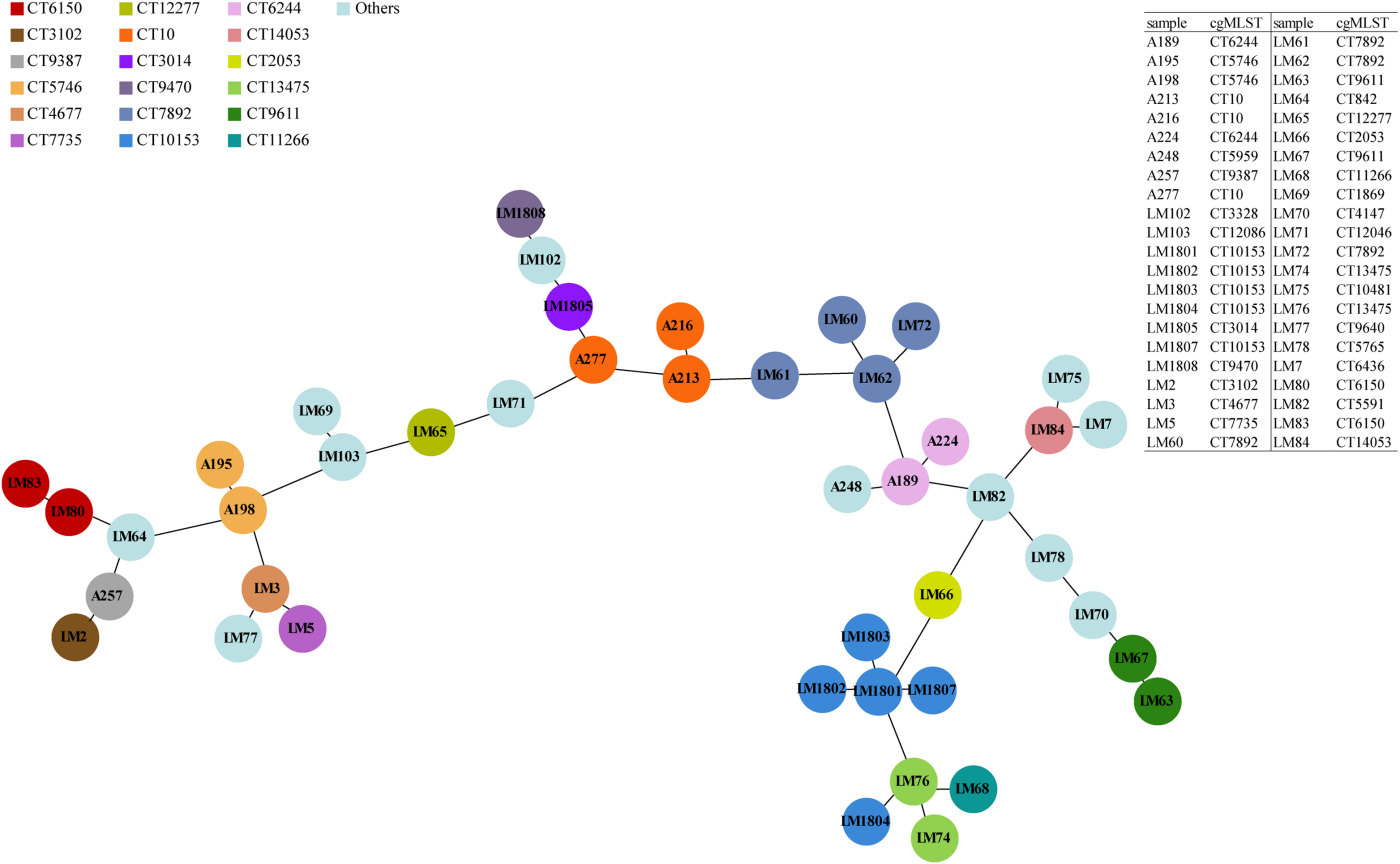
Phylogenetic relationships of *L. monocytogenes* strains as illustrated by a Minimum Spanning Tree. The minimum spanning tree is based on cgMLST and represents the relationships among 44 *Lm* strains with 30 distinct CTs. Each circle symbolizes an isolate, colored according to its specific CT (referenced in the key at the top left). Links between circles denote phylogenetic distances between isolates.

To achieve high-resolution species typing results, cgMLST analysis was carried out and the minimum spanning tree was drawn based on 1748 core gene loci (**Figure 1**). These 44 *Lm* strains were divided into 30 CTs. The CT10153 and CT7892 were the dominant types, while the CT10153, CT13475, and CT11266 had a relatively close genetic evolutionary distance, and the strains of CT10153 and CT13475 were all particularly isolated from imported food products from Vietnam at different time periods. The 10 strains collected from China between 2012 and 2014 exhibited distant genetic relationships, suggesting that the spread of *Lm* strains had also become more widespread over time. Moreover, LM63, belonging to none of existing STs in MLST analysis, was classified into the same type of CT9611 with LM67 strain in the cgMLST analysis.

### 3.3 Genomic elements and phylogeny of 44 *L. monocytogenes*

Mobile genetic elements (MGEs) are potent drivers of genome evolution (Matereke and Okoh, 2020). To identify the impact of MGEs on *Lm* genome evolution, it was attempted to analyze the relationship of *Lm* evolution with the number of prophages, CRISPRs, and ISs in genome.

Prophages are a class of MGEs that are ubiquitous in bacterial genomes, and they also mediate transduction process causing the horizontal transfer of genetic materials between bacteria, including toxicity and antibiotic resistance genes (Vu et al., 2021). In the present study, prophages with the predictive values in the genomes of 44 *Lm* strains were predicted using the PHASTER database. Higher values indicated a greater likelihood of the fragment being incorporated into intact phages. The number of intact prophages in each sample is presented in **Appendix Table 1**. The majority of these prophages were *Lm* phages.

The CRISPR system is an acquired immune system that is widely existed in bacteria. It plays an important role in maintaining the stability of bacterial genome. The CRISPR system was predicted using MinCED (Ver. 0.2.0), and the CRISPR system was identified in 41 *Lm* samples. The relevant statistics are summarized in **Appendix Table 2**.

ISs are important genetic elements that drive the evolution of bacterial genome and lead to the instability of genome. In the present study, ISs were predicted through the BLAST (evaluate = 1-5) and ISfinder databases. Notably, ISs were predicted in all 44 *Lm* samples, while only 18 samples had complete ISs. The number of ISs is presented in **Appendix Table 3**. Most of these ISs were from *Lm*, and a limited number of them were from *Leucanostoc mesenteroides*. Among them, there was only one encoding gene of transposase, and no other functional elements were involved.

**Figure 2:**
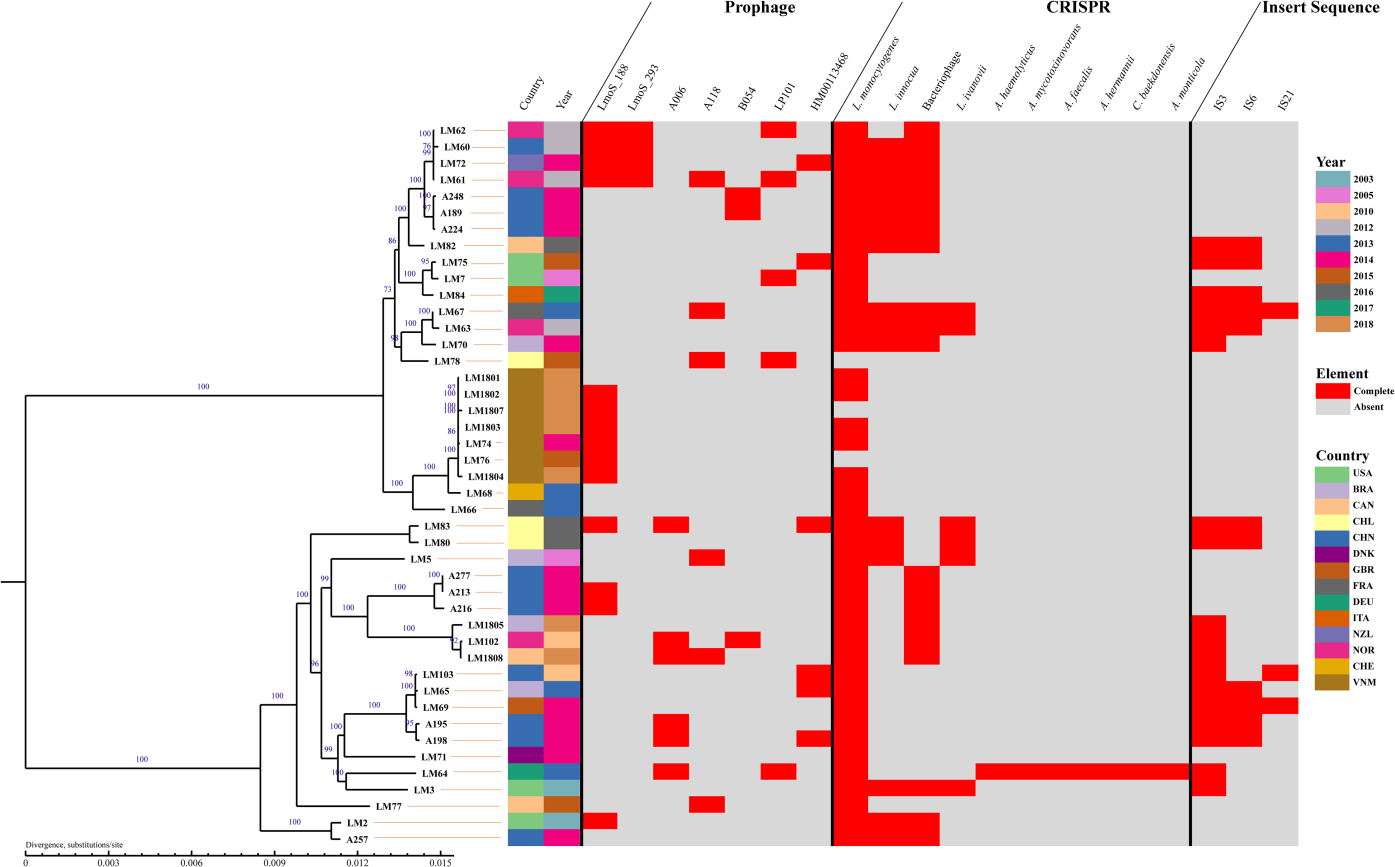
Phylogeny and mobile genomic elements of 44 *L. monocytogenes* strains. Using the RAxML software, a phylogenetic tree was constructed from gene sequences of identified homologous genes. Depicted within the tree are complete prophages, CRISPR, and IS identified in each strain. Country abbreviations: CHN-China, USA-the United States, BRA-Brazil, DNK-Denmark, DEU-Germany, FRA-France, CAN-Canada, NOR-Norway, CHE-Switzerland, NZL-New Zealand, ITA-Italy, GBR-the United Kingdom, VNM-Vietnam, CHL-Chile.

It was reported that MGEs are ideal loci for bacteria typing and evolutionary analysis (Frost et al., 2005; Botelho et al., 2019; Weisberg and Chang, 2023). In the present study, a phylogenetic tree was constructed by RAxML based on single-copy gene. The results revealed that 44 *Lm* strains were mainly classified into two lineages (**Figure 2**). One lineage consisted of 20 strains, spanning from A257 to LM83, while the remaining strains were in the other lineage.

### 3.4 The whole-genome-based SNP phylogenetic analysis

Phylogeny is utilized to analyze the relationship among organisms, and it is widely applied in phylogenetic classification, epidemiology, and ecology (Lüth et al., 2020). To identify the epidemiological characteristics, the analysis was carried out based on SNP of these *Lm* strains at different time periods and from different countries. It was found that there were numerous SNP sites in 44 *Lm* strains.

**Figure 3:**
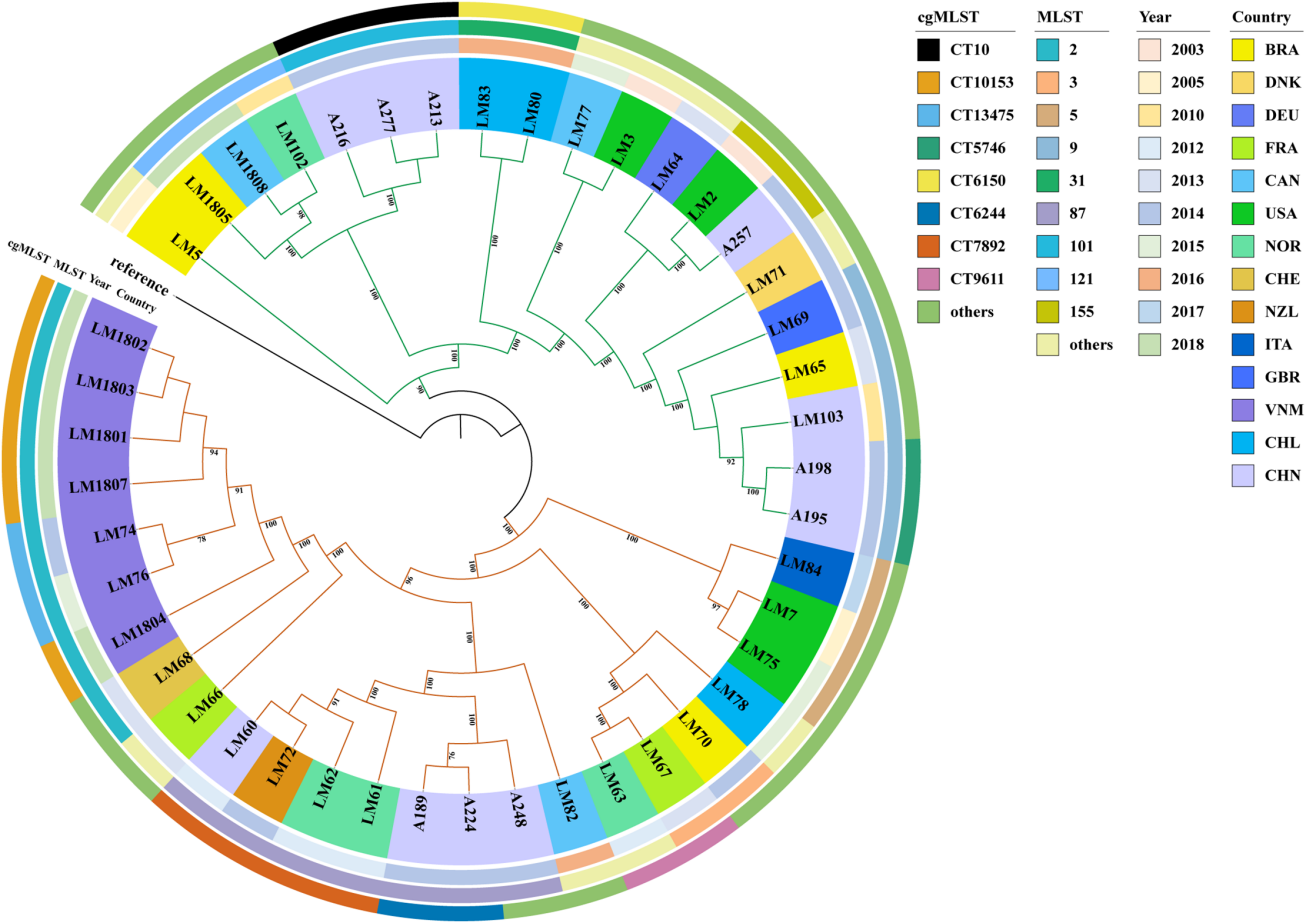
SNPs-based phylogenetic tree. The maximum-likelihood phylogenetic tree was established using34,128 SNPs called from 44 *Lm* strains. The reference strain was *Lm* GCA_900187225.1 published by Sanger sequencing center in the UK. All isolates were clustered into two distinct clades. The color strips in outer circle represent different cgMLST genotypes, those in middle circle represent different collecting time (year), and those in inner circle represent different collecting regions.

Using *Lm* genome assembly GCA_900187225.1 published by the Sanger sequencing center in the UK in 2017 as the reference strain (Kremer et al., 2017), the 44 *Lm* strains were subsequently subjected to the whole-genome-based SNP phylogenetic analysis. These 44 strains could be roughly classified into two clades (**Figure 3**), in which the strains from LM5 to A195 belonged to the first clade, and the remaining strains were categorized as the second clade. The second clade had significantly more strains, whereas there were some differences between strains. In order to explore the reasons related to these differences, the evolutionary tree was annotated according to the time of collection and geographical regions of the strains.

Significant differences were found in the time of *Lm* strain collection between the first and second clades. In the first clade, most of *Lm* strains were obtained in earlier. The *Lm* strains in the second clade were mainly collected from 2010 and later. The LM5 strain obtained in 2005 was the most similar to the reference.

Additionally, the characteristics of bacteria collected from different regions were analyzed in the phylogenetic tree. The relationship between *Lm* strains from Vietnam was relatively close, and there was also a certain relationship between *Lm* strains spanning 11 years (from 2003 to 2014) from other countries. In the first clade, the bacterial strains were collected over an approximately 11-year period, ranging from 2003 to 2014, and strains from the same regions exhibited similarity. This indicated that there was a mutual relationship between *Lm* strains from different time periods or regions.

### 3.5 Analysis of virulence genes

**Figure 4:**
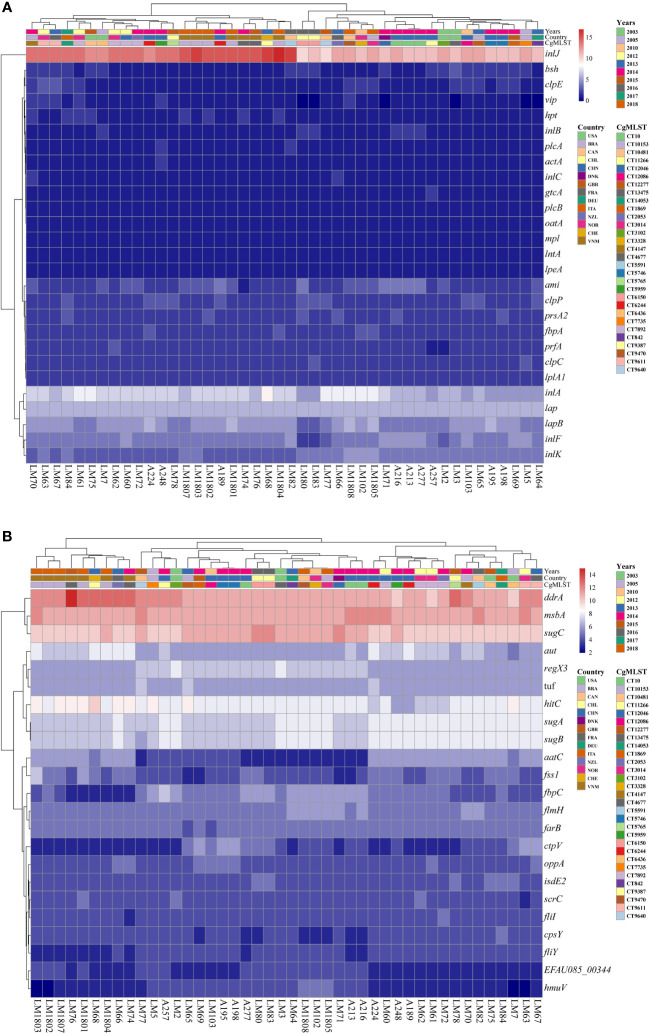
Virulence factor analysis results. Heatmap and hierarchical clustering was performed based on gene copies of virulence factors common across the *Lm* strains **(A)** or virulence factors with high abundance across our dataset **(B)** using Euclidean distance measurement method, and was visualized with the “pheatmap” package of R. The color key indicates the abundance of the virulence genes. Red: high abundance, Blue: low abundance.

Virulence factors produced by pathogens play an crucial role in the occurrence of diseases (Wartha et al., 2023). To evaluate the virulence of these *Lm* strains, the virulence factors were identified through analyzing the genome sequences of these 44 strains in the VFDB (Chen et al., 2005; Matto et al., 2022). It was revealed that the majority of the known virulence factors associated with *Lm* were present in the 44 *Listeria* strains, *inlJ* was the richest one, and they were also rich in *inlA* and *lap* (**Figure 4A**). In *Lm*, *inlJ* and *lap* were associated with cell adhesion, and *inlA* was correlated with bacterial migration (Osek and Wieczorek, 2022). Meanwhile, 6 strains (LM3, LM5, LM64, LM71, LM80, and LM83) did not contain *vip* virulence gene. Moreover, it was found that some other common virulence factors did not exist in the 44 *Lm* strains, including the exoenzyme SMase, the *pgdA* gene related to immune evasion, the *auto*, *inlP*, and *p60* genes in migration, the *svpA* gene in iron ion absorption, the peptidase Lsp, and the *LLO* and *LLS* encoding genes related to toxin.

In addition to the known virulence factors associated with *Lm*, the dataset exhibited an enrichment of other factors compared with VFDB. The heatmap showed that these 44 strains had the highest abundance of *ddrA*, *msbA*, and *sugC* genes, followed by *hitC*, *sugA*, and *sugB* genes (**Figure 4B**). Interestingly, the *ddrA* gene exhibited a higher copy number mainly in the strains from Vietnam, compared to strains derived from other countries/regions. However, the roles of these genes were not reported, and further studies are required to clarify their roles in *Listeria*.

## 4 Discussion

In the present study, 49 bacterial strains were collected from food samples originating from 14 countries/regions spanning 15 years. Their genomic DNA was sequenced using high-throughput sequencing technology. Among them, five isolated strains (A212, LM6, LM79, LM4, and LM1806) were found to be contaminated and were therefore excluded from further analysis. As a result, a total of 44 *Lm* strains were analyzed in terms of their genomic sequences to investigate the bacterial genotypes and SNPs. MLST is a widely utilized method for bacterial genome typing based on DNA sequences. It was initially developed in 1998 and has been used to discern natural genetic variations in *N. meningitidis* (Maiden et al., 1998). In contrast to traditional molecular biology genotyping methods, MLST possesses higher resolution and the ability to readily distinguish bacterial subtypes, highly facilitating the exploration of the relationship between bacterial subtypes and diseases. Currently, a huge amount of MLST data related to *Listeria* are stored in databases. In the present study, MLST typing analysis was conducted on the genomic sequence data of 44 *Lm* strains. As indicated in **Table 1**, it was found that the *Lm* strain LM63, isolated from Norway in 2012, had an unknown typing result due to the presence of new SNP information in the *dat* gene, a gene essential for D-alanine biosynthesis. D-alanine, not synthesized by vertebrates, is a crucial component required for bacterial cell wall synthesis and growth. While the deletion of the *dat* gene did not exhibit reversion or significant suppression of its auxotrophy under laboratory conditions, it alleviated the toxicity of *Lm* and could potentially serve as an attenuated vaccine (Zhao et al., 2005). This suggests that mutations in the *dat* gene may play a crucial role in regulation of *Lm* function. Although there were differences in the typing of other strains except for LM63, they were relatively centralized in genotype.

Subsequently, these genomic data were utilized to construct phylogenetic trees, and the evolutionary relationships between these isolated *Lm* strains from different regions and at different time periods were analyzed. Notably, SNPs, as polymorphisms in genomic sequences, are highly appropriate for survival analysis in the context of evolution (Hammarlof et al., 2018; Losic et al., 2020). In the present study, it was found that these 44 *Lm* strains had different SNP loci, and a phylogenetic tree was constructed based on these SNPs. Compared with the reference genome sequence, the 44 *Lm* strains were roughly divided into two clades. There was a difference in sampling time between strains in the first clade and those in the second clade. In addition, *Lm* strains obtained in earlier years were mainly concentrated in the first clade. Strains in the second clade were mostly collected from 2010 onwards. The strain LM5, obtained in 2005, displayed the highest similarity to the reference genome.

Research has indicated that the evolution of bacteria follows distinct biogeographic patterns (Martiny et al., 2006; Li and Hu, 2021; Gao et al., 2023). Huang et al. discovered the distinct evolutionary trajectories of the spread of *E. coli* ST1193 in China and abroad, and this was confirmed by the hybrid analysis of core genome and accessory genome phylogeny, virulence factors, antibiotic resistance genes, and plasmid replicon profiles (Huang et al., 2021). Similar results were obtained in the present study. It was found that *Lm* strains isolated from Vietnamese products clustered well together in the minimum spanning tree analysis, SNP-based evolutionary analysis, single-copy gene-based evolutionary analysis, mobile element analysis (including prophages, ISs, CRISPRs), and virulence gene analysis. Moreover, the results of evolutionary analysis indicated that these strains were different from others isolated from products of other countries/regions. Furthermore, *Lm* strains isolated from Chilean products were also relatively clustered together. These findings indicated that *Lm* had been evolved independently in specific geographical regions.

Additionally, it was found that the evolutionary relationships between *Lm* strains from Vietnam were relatively close. Furthermore, it was noted that the genetic relationships between *Lm* strains from different countries/regions were relatively close, which not only existed within the same continents, such as between the United States and Canada (LM77 and LM3), France and Norway (LM67-1 and LM63), but also found between intercontinental countries, including China and Brazil, China and the United States, China and New Zealand, Vietnam and Sweden, Vietnam and France, and the United States and Italy. The transmission of *Lm* strains between different regions might be achieved through direct vertical or horizontal gene transfer. According to the results of evolutionary analysis and the isolated time of *Lm*, it was hypothesized that strains existed in Vietnam might be spread to Vietnam through food trade after their transmission within Europe. This might be attributed to the biological characteristics of *Lm*, which is existed ubiquitously in the nature, it can survive and reproduce even at low temperatures, and it can easily spread into food processing environments and cause food contamination. Nonetheless, these findings are insufficient to rule out the effects of other traded products on the spreading of *Lm*. Moreover, it was revealed that there were certain similarities among strains from different regions spanning 11 years (from 2003 to 2014), suggesting that there might be possible transmission events of *Lm* strains between different sources and time periods.

Finally, the virulence genes involved in these 44 *Lm* strains were analyzed. Virulence is described as an ability of an organism to infect the host and cause a disease. Virulence factors, which are either secretory, membrane associated or cytosolic in the nature, comprise a group of molecules produced by pathogenic microorganisms that may cause disease in the host (Sharma et al., 2017). The VFDB database was established by the Institute of Pathogenic Microbiology, Chinese Academy of Medical Sciences, which is an integrated and comprehensive online database for curating information related to virulence factors of bacterial pathogens, and it is widely recognized due to its up-to-date status (Chen et al., 2005). In the VFDB database, there were 36 major virulence factors identified in *Lm*, including actin-based motility, adherence, bacterial cell wall modification, bile resistance, exoenzyme, immune evasion, immunomodulator, intracellular growth, invasion, iron uptake, metabolic adaptation, peptidase, regulation, stress protein, and toxin (Chen et al., 2005). In the present study, VFDB database was employed to annotate the virulence factors of the 44 *Lm* strains. The results showed that the 44 strains were all enriched in genes, such as *inlJ*, *inlA*, and *lap*. Besides, *inlJ* and *lap* genes were related to cell adhesion, and *inlA* gene was related to bacterial migration. These results demonstrated that there were similarities in virulence genes among different strains.

In the analysis of common virulence genes, it was indicated that the *Lm* strains contained the consistent compositions of virulence genes. This demonstrates that there might be no significant differences in human diseases caused by *Lm* infection. Among isolated strains, only 6 strains (LM3, LM5, LM64, LM71, LM80, and LM83) did not contain *vip* virulence gene, which might be attributed to random gene loss as there was no association between the time and region of their isolation. Prior researches have shown that *vip* gene related to migration did not exist in all nonpathogenic *Listeria* species as well as in particular in two rare *Lm* serovars (4a, 4c) (Doumith et al., 2004; Cabanes et al., 2005). Multiple studies have found that knocking out *vip* gene in *Lm* had no significant effect on the bacterial growth. These results indicated that *vip* gene may have other functions in the growth of *Lm*. Nevertheless, there were significant difference in the gene copy number of common virulence genes among these isolated *Lm* strains, especially in *inlJ* gene, which encoded internalin composed of 916 amino acids and presented only in *Lm*. Those *inlJ* gene knocking-out strains exhibited a lower invasive ability of *Lm* (Sabet et al., 2005), and this gene was missed in avirulent *Listeria* species (Liu et al., 2003; Doumith et al., 2004). Accordingly, the isolated strains could be classified into two distinct groups based on the copy number of the *inlJ* gene, suggesting that these strains might present significant differences in disease severity. However, there is no study concentrating on the relationship between the copy number of the *inlJ* gene and the disease severity, and this should be investigated in the future studies.

Notably, it was found that the copy number of virulence genes, such as *inlJ* and *ddrA* genes, in *Lm* strains isolated from Vietnam, and their closely related strains isolated from Sweden and France, was significantly higher than that from other regions. This not only indicated that there were specific biogeographical characteristics in *Lm* evolution and the possibility of *Lm* cross regional transmission, but also suggested the likelihood of cross-regional trade, leading to outbreak of foodborne diseases caused by *Lm* infection in other regions.

According to the proposed comprehensive monitoring system for imported products carried *Lm*, a reliable relationship was explored between foodborne diseases and potential sources. Given the feasibility of tracking imported food products, it becomes possible to promptly trace the origins of foodborne pathogenic strains responsible for disease outbreaks. By leveraging traceability results, appropriate measures can be taken, drawing from the treatment experience in the source areas. This approach allows for the rapid containment of  foodborne diseases, thereby mitigating the associated social and economic losses. Therefore, the characterization of the foodborne pathogen *Lm* might play a crucial role in preventing further infections through rapid identification and precise allocation of contaminated food sources.

## Data availability statement

The data presented in the study are deposited in the NCBI Bioproject repository, accession number PRJNA1036137..

## Author contributions

LZ: Data curation, Formal analysis, Methodology, Software, Visualization, Writing – original draft. XJ: Data curation, Formal analysis, Writing – original draft. YW: Data curation, Formal analysis, Writing – review & editing. WX: Resources, Writing – review & editing. FW: Conceptualization, Funding acquisition, Methodology, Project administration, Supervision, Writing – review & editing. XH: Conceptualization, Funding acquisition, Project administration, Supervision, Writing – review & editing.
